# Beyond Tumor Volume: An Integrated Radiological Model of Tumor Load, Anatomical Spread, and Mass Effect for Survival Prediction in Adult Grade 4 Diffuse Astrocytic Tumors

**DOI:** 10.3390/medicina62050959

**Published:** 2026-05-14

**Authors:** Mustafa Emre Sarac, Zeki Boga, Ali Arslan, Ümit Kara, Mehmet Ozer, Ali Harmanoğullarından, Ali Sürmelioğlu, Feryal Karaca, Zişan Nur Sürmelioğlu, Yurdal Gezercan

**Affiliations:** 1 Department of Neurosurgery, Adana City Training and Research Hospital, Adana 01230, Turkey; zekiboga2013@gmail.com (Z.B.); aliarslan26062006@hotmail.com (A.A.); aliharmanoglu@gmail.com (A.H.); ali.surmeli.30@gmail.com (A.S.); gezercan@hotmail.com (Y.G.); 2 Department of Anesthesiology, Adana City Training and Research Hospital, Adana 01230, Turkey; doctorumit@gmail.com; 3 Department of Neurosurgery, Niğde Training and Research Hospital, Niğde 51100, Turkey; mehmetozerdr@gmail.com; 4 Department of Radiation Oncology, Adana City Training and Research Hospital, Adana 01230, Turkey; feryalkaraca@gmail.com; 5 Department of Radiology, Adana City Training and Research Hospital, Adana 01230, Turkey; elazisan@gmail.com

**Keywords:** glioblastoma, astrocytoma, magnetic resonance imaging, brain neoplasms, prognosis, survival analysis, edema

## Abstract

*Background and Objectives:* Prognostic assessments in grade 4 diffuse astrocytic tumors primarily depend on clinical and molecular characteristics, with radiological attributes frequently assessed in isolation. In this study, we explored whether an integrated radiological approach combining tumor burden, anatomical spread, and mass effect could contribute to survival prediction. *Materials and Methods:* A total of 310 adult patients with histopathologically confirmed grade 4 diffuse astrocytic tumors, diagnosed between January 2022 and January 2025, were included in this retrospective single-center cohort. Preoperative MRI was used to assess contrast-enhancing tumor volume, edema volume, and brain volume, combined with anatomical spread and midline shift as a marker of mass effect. Tumor burden was defined as the ratio of enhancing tumor volume to brain volume (ETV/BV). Overall survival was analyzed using Kaplan–Meier and Cox regression methods. Model performance was evaluated with the C-index, bootstrap internal validation, and 12-month calibration. *Results:* Tumor burden was higher in IDH-wildtype tumors, which also showed higher midline shift and more frequent deep structure involvement and contralateral extension. In multivariable analysis, IDH status, age, tumor burden, midline shift, and deep structure involvement were independently associated with overall survival. A greater tumor burden was associated with reduced survival. The addition of molecular and imaging-derived variables improved discrimination, increasing the C-index from 0.69 to 0.76. Following bootstrap validation, the corrected value was 0.73. Calibration at 12 months demonstrated acceptable agreement between predicted and observed outcomes. *Conclusions*: An integrated radiological approach that combines tumor burden, anatomical spread, and mass effect may support prognostic assessment in addition to established clinical and molecular variables.

## 1. Introduction

Adult grade 4 diffuse astrocytic tumors, including glioblastoma and IDH-mutant astrocytoma, are associated with poor survival despite current multimodal treatment approaches [1,2]. The standard treatment protocol generally includes surgery, radiation therapy, and temozolomide [3,4]. Nevertheless, many patients develop early disease progression, and long-term survival is uncommon [3]. The clinical course and treatment response of these tumors vary widely, and patients receiving similar therapies may still experience markedly different survival outcomes [2,5]. Consequently, identifying reliable prognostic markers at the time of diagnosis is vital for guiding treatment decisions and improving patient management [2,5].

Prognosis in grade 4 diffuse astrocytic tumors is determined by a combination of factors, including the patient’s age, their neurological condition when first diagnosed, molecular markers, and the treatment used [2,5]. Older age, poor performance status, and limited surgical removal of the tumor are associated with worse survival outcomes [5,6]. The prognostic value of molecular markers has become increasingly acknowledged in contemporary research. Specifically, the occurrence of isocitrate dehydrogenase (IDH) mutations has been demonstrated to substantially influence the clinical trajectories of high-grade gliomas [7]. Tumors characterized by IDH mutations are typically associated with improved prognoses and a more gradual progression; in comparison, IDH-wildtype glioblastomas frequently exhibit a more aggressive clinical course [5]. Most prognostic models are still based mainly on clinical and molecular variables, and imaging findings are usually included in a more limited and less standardized way [5,8,9].

Magnetic resonance imaging (MRI) is the primary imaging modality used in the diagnosis, treatment planning, and follow-up of grade 4 diffuse astrocytic tumors. It is crucial for assessing the structure of tumors and the extent of their spread [8]. The results of recent studies demonstrate that imaging features can provide important information for both diagnosis and outcome prediction [8,9]. Specifically, volumetric assessments, including enhancing tumor volume and total tumor volume, have been linked to overall survival in several cohort studies [9,10]. In many studies, tumor burden is assessed using absolute volume; in contrast, other radiological features, such as spread and mass effect, are considered independently [11,12,13,14,15].

Moreover, differences in brain volume between individuals could potentially influence clinical results in patients with similar tumor sizes in this setting. Beyond tumor size, the manner in which the tumor disseminates within the cranial cavity, along with the resultant mass effect, may significantly influence the trajectory of the disease [11,12,13]. While tumor volume, anatomical spread, and mass effect have each been associated with clinical outcomes, these parameters are typically evaluated in isolation, and integrated radiological approaches that combine these features within a unified prognostic framework remain limited [8,9,16].

In this study, we move beyond simple volumetric measures by using normalized tumor burden—defined as the ratio of enhancing tumor volume (ETV) to brain volume (BV) (ETV/BV)—combined with anatomical spread and mass effect in adult patients with grade 4 diffuse astrocytic tumors. Accordingly, the aim of this study is to develop an integrated radiological assessment framework that combines normalized tumor burden with parameters reflecting anatomical spread and mass effect, and to evaluate their independent and combined impact on overall survival, as well as their contribution to the performance of a prognostic model.

## 2. Methods

### 2.1. Study Design and Patient Selection

**Figure 1:**
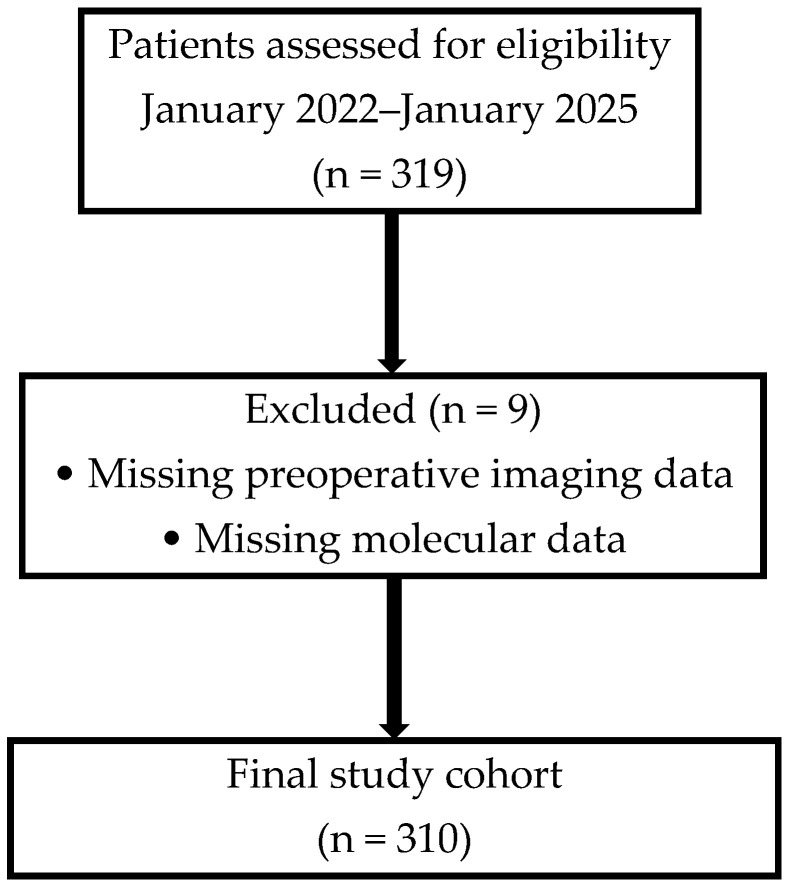
Flowchart of patient selection, including screening, exclusion, and final study cohort.

In this study, we employed a retrospective, single-center cohort design. The study included patients diagnosed at our institution between 1 January 2022 and 1 January 2025. Patients diagnosed during the study period and who met the inclusion criteria were enrolled in the study in the order in which they were identified. All patients were followed until 1 January 2026, which was defined as the administrative censoring date. Patients aged 18 years or older with histopathologically confirmed grade 4 IDH-mutant astrocytoma or glioblastoma were included. Availability of preoperative magnetic resonance imaging at the time of diagnosis was also required for inclusion. Patients with recurrent disease, a prior diagnosis of low-grade glioma, previous cranial radiotherapy or chemotherapy, or missing preoperative imaging data were excluded. A total of 319 patients were screened during the study period. Nine patients with missing imaging or molecular data were excluded, resulting in a final analysis cohort of 310 patients. The rate of missing data was 2.8% of the initial group. Therefore, statistical analyses were performed using the final dataset. The baseline demographic and clinical characteristics of excluded patients were compared with those of the final analysis cohort, and no significant differences were observed. The patient selection process is summarized in Figure 1.

### 2.2. Histopathological and Molecular Assessment

Histopathological evaluation was performed in the pathology department of our institution. Tumor specimens were fixed in 10% neutral buffered formalin for 12–24 h and then processed routinely and embedded in paraffin. Sections approximately 3–5 μm thick were prepared and stained with hematoxylin and eosin (H&E) for morphological assessment. Immunohistochemical staining was performed as part of routine diagnostic work-up using markers applied in glioma evaluation, including IDH1 (R132H), ATRX, and p53. Staining procedures were carried out on an automated immunohistochemistry platform following standard laboratory workflows. Isocitrate dehydrogenase mutation status was first evaluated by immunohistochemistry. In cases with negative or equivocal staining results, additional sequencing-based molecular testing was performed to clarify IDH status. Tumors were classified according to the 2021 WHO Classification of Tumors of the Central Nervous System.

### 2.3. Radiological Assessment and Parameter Definitions

Preoperative magnetic resonance images obtained at the time of diagnosis were reviewed retrospectively. All images were analyzed in DICOM format using dedicated imaging software (RadiAnt DICOM Viewer, version 2024.1; Medixant, Poznań, Poland). The evaluations were performed by a radiologist who was blinded to clinical outcomes and survival data. Deep structure involvement was defined as direct infiltration of the basal ganglia, thalamus, internal capsule, or brainstem. Midline shift was measured in millimeters as the maximum displacement from the midline at the level of the septum pellucidum. Contralateral extension, ventricular contact, and multifocality were recorded separately. Contralateral extension was defined as tumor spread across hemispheres through the corpus callosum, and multifocality as the presence of at least two distinct tumor foci.

Tumor and peritumoral edema volumes were calculated using semi-automatic segmentation software (3D Slicer, version 5.6.2; Brigham and Women’s Hospital, Boston, MA, USA). Enhancing tumor volume was derived from contrast-enhanced T1-weighted images. Three-dimensional segmentation was applied to delineate contrast-enhancing tumor regions, including areas of necrosis. Peritumoral edema volume was defined based on hyperintense signal abnormalities on T2/FLAIR sequences. Brain parenchymal volume was defined by excluding ventricular and sulcal cerebrospinal fluid spaces and was calculated using FSL (FMRIB Software Library, version 6.0.7; Oxford Centre for Functional MRI of the Brain, Oxford, UK) with the SIENAX tool. Automated segmentation results were manually refined when necessary, and segmentation accuracy was visually confirmed in all cases. All volumes were recorded in cubic centimeters (cm^3^). To account for differences in brain size, the ETV/BV and EV/BV ratios were calculated and then analyzed as continuous variables.

### 2.4. Data Collection

Clinical and radiological data were retrospectively obtained from the institutional electronic medical records and imaging archives. Clinical variables included age at diagnosis, sex, the type of surgical intervention, and adjuvant therapies. Surgical approaches were classified as biopsy, subtotal resection, or gross total resection. Adjuvant treatments, including radiotherapy and temozolomide, were documented. A previously established standardized data collection tool was used to gather information on volumetric and anatomical tumor parameters. To assess the reliability of the radiological evaluations, imaging data from 40 randomly selected cases were re-evaluated by an external neurosurgeon who was blinded to clinical outcomes. Interobserver agreement was assessed using Cohen’s kappa coefficient for categorical variables and the intraclass correlation coefficient (ICC) based on a two-way random-effects model with absolute agreement for continuous variables. After merging all clinical and radiological data, statistical analyses were performed on the final dataset.

### 2.5. Treatment Protocol

Treatment was determined according to the standard neuro-oncology practice at our institution. We aimed to achieve maximal safe resection where feasible, with biopsy performed in cases where safe resection was not possible. Surgical procedures were recorded as biopsy, subtotal resection, or gross total resection.

Postoperative adjuvant therapy consisted of radiotherapy and temozolomide administered according to the standard Stupp protocol in clinically eligible patients. Radiotherapy was delivered in fractions to a total dose of approximately 60 Gy and was administered concurrently with temozolomide, followed by adjuvant temozolomide therapy in appropriate patients.

### 2.6. Follow-Up and Outcome Measures

The primary outcome of the study was overall survival. Overall survival was defined as the time from the date of baseline magnetic resonance imaging performed at diagnosis to the date of death. All radiological analyses were based on this baseline imaging. Patients who were alive at the end of follow-up were censored at the date of the last known clinical evaluation. Survival data were obtained from institutional electronic medical records and outpatient follow-up records and were verified using the national death registry.

### 2.7. Statistical Analysis

Statistical analyses were performed using IBM SPSS Statistics for Windows (version 26.0; IBM Corp., Armonk, NY, USA) and R software (version 4.3.2; R Foundation for Statistical Computing, Vienna, Austria). The distribution of continuous variables was assessed using visual methods such as histograms and Q–Q plots. Variables with a normal distribution were presented as mean ± standard deviation (SD), as appropriate. In contrast, variables that did not follow a normal distribution were presented as the median and interquartile range (IQR). Categorical variables were reported as counts and percentages.

Group comparisons for categorical variables were performed using the chi-square test or Fisher’s exact test, as appropriate; in comparison, continuous variables were analyzed with Student’s *t*-test or the Mann–Whitney U test according to their distribution. Overall survival was estimated with the Kaplan–Meier method, and group differences were evaluated using the log-rank test. Death was defined as the event of interest, and the follow-up period commenced on the date of the initial MRI.

Using the maximally selected log-rank statistic, the best cut-off points were determined for continuous variables that represented normalized tumor burden, specifically ETV/BV and EV/BV. A series of candidate cut-off values was evaluated, and the threshold yielding the highest log-rank statistic was selected as optimal. To reduce the risk of overfitting, the selected cut-off values were interpreted in conjunction with their clinical relevance. These thresholds were used for survival stratification in Kaplan–Meier analyses, whereas continuous forms of the variables were retained in the multivariable Cox regression models to preserve model robustness.

Cox proportional hazards regression models were used to evaluate how prognostic factors affected overall survival. Initially, candidate variables were assessed using univariable Cox regression analysis. Variables considered clinically relevant and those with *p* < 0.10 in univariable analysis were included in the multivariable model. Treatment-related variables were not retained in the final model to minimize confounding by indication and to more effectively isolate the independent prognostic value of radiological features. Hazard ratios (HR) and their associated 95% confidence intervals (CI) were reported. The proportional hazards assumption was validated through examination of Schoenfeld residuals.

Continuous variables were analyzed in their original form, and ETV/BV and EV/BV ratios were included as independent variables in the models. Spearman correlation coefficients were used to assess the relationships between the volume variables. The variance inflation factor (VIF) was used to evaluate multicollinearity. To ensure model stability, model complexity was managed by maintaining an events-per-variable (EPV) ratio of no less than 10. For model performance, a reference model based on age and treatment-related variables was used, and its performance was then compared with a model that additionally included molecular status and imaging-derived variables. The final multivariable Cox model constituted the prognostic model. Model discrimination was assessed through Harrell’s concordance index (C-index). Internal validation was achieved via bootstrap resampling, which involved 1000 iterations, and optimism-corrected C-index values were then calculated. Model calibration was assessed using calibration plots based on bootstrap resampling (1000 iterations) at a prespecified time point. Using the regression coefficients obtained from the multivariable model, a personalized prognostic index, also known as a risk score, was computed for every patient. Patients were then categorized into three equal tertiles, based on the distribution of these risk scores, thereby establishing low-, intermediate-, and high-risk categories. Statistical significance was determined using a two-sided *p*-value of less than 0.05.

**Figure 2:**
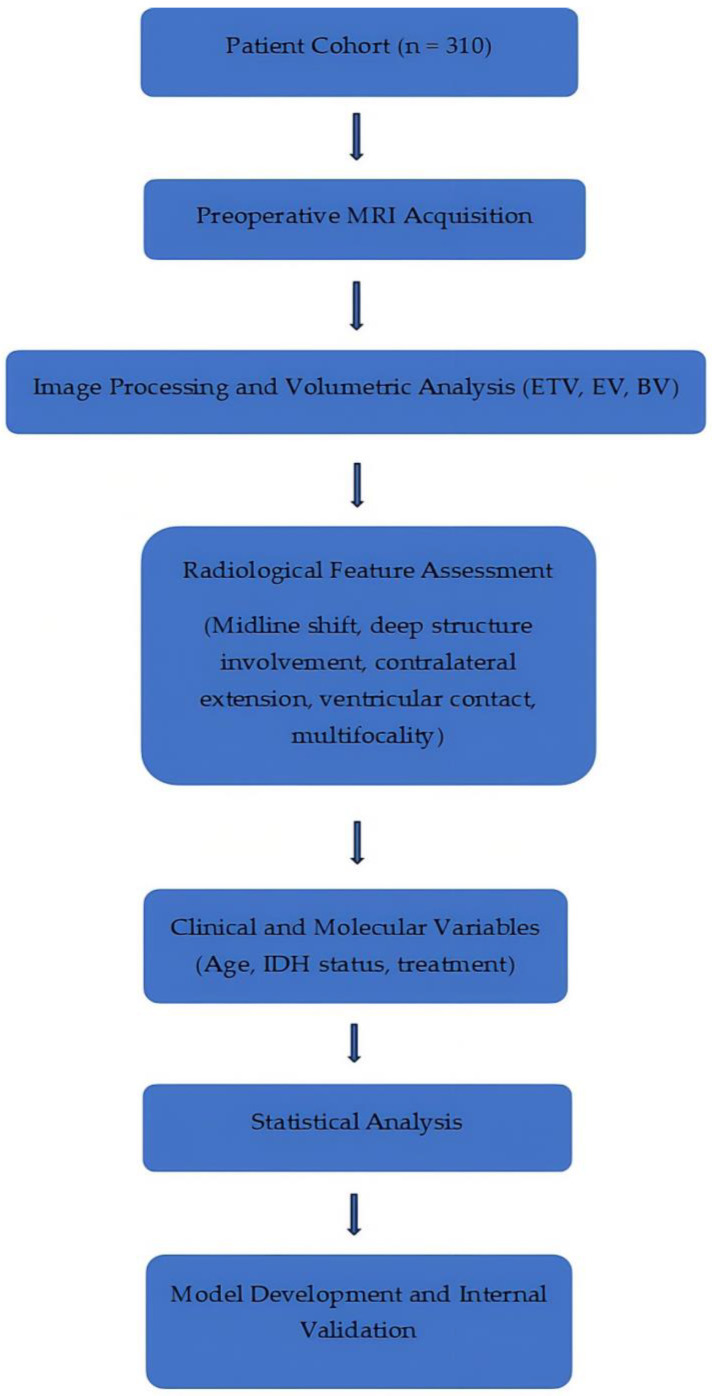
Schematic overview of the study workflow and analytical pipeline. The diagram summarizes the main steps of the study.

To provide an overall view of the study design and analytical approach, the workflow of the study is summarized in Figure 2. This schematic representation integrates the main steps, including image processing, radiological and clinical variable assessment, and statistical analysis.

### 2.8. Ethical Approval and Informed Consent Statement

This study was conducted in accordance with the Declaration of Helsinki and approved by the Clinical Research Ethics Committee of Adana City Training and Research Hospital (Meeting No: 24; Decision No: 1258; Approval Date: 30 March 2026). Due to the retrospective design, the requirement for additional study-specific informed consent was waived by the ethics committee. Written informed consent permitting the use of anonymised clinical data for research purposes had been obtained from all patients or their legal representatives in accordance with institutional regulations.

## 3. Results

### 3.1. Study Population and Baseline Clinical and Radiological Characteristics

The final analysis included 310 patients, of whom 82 were alive at the end of the follow-up period and were censored in the survival analysis. Based on the 2021 World Health Organization classification of central nervous system tumors, 96 patients (31.0%) were classified as having astrocytoma, IDH-mutant, CNS WHO grade 4, and 214 patients (69.0%) were classified as having glioblastoma, IDH-wildtype, CNS WHO grade 4. No significant differences were found in baseline demographic and clinical characteristics between the patients excluded due to missing data and those included in the analysis. When molecular subgroups were compared, patients with IDH-wildtype tumors were found to be significantly older. In addition, parameters representing normalized tumor burden were higher in this group. In particular, ETV/BV and EV/BV ratios, as well as midline shift, were higher, whereas contralateral extension and deep structure involvement were more frequently observed in the IDH-wildtype group. Re-evaluation of 40 randomly selected cases yielded a Cohen’s kappa coefficient of 0.73 for categorical radiological variables. For continuous measurements, the intraclass correlation coefficient (ICC) was 0.87. The detailed distribution of demographic, treatment-related, and radiological characteristics of the study population is presented in Table 1.

### 3.2. Overall Survival According to Molecular Subtype

Overall survival differed significantly between molecular subtypes. Survival was longer in patients with IDH-mutant tumors; in comparison, survival was markedly shorter in the IDH-wildtype glioblastoma group (log-rank *p* < 0.001).

### 3.3. Tumor Burden Analysis and Survival

**Figure 3:**
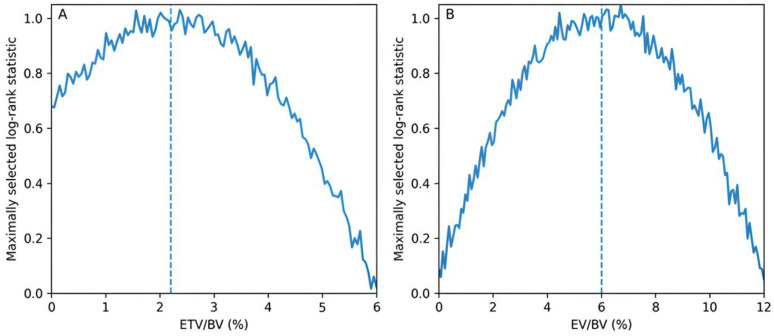
Determination of cut-off values for parameters representing normalized tumor burden. (**A**) The threshold value showing the strongest association with overall survival for the ETV/BV ratio is presented; the selected cut-off value is 2.2%. (**B**) The corresponding analysis for the EV/BV ratio is shown; the selected cut-off value is 6.0%. The curves represent the log-rank statistic calculated for each possible cut-off value, and the dashed vertical lines indicate the selected threshold values.

Optimal cut-off values providing the strongest discrimination for survival were determined for parameters representing normalized tumor burden. The cut-off value was 2.2% for the ETV/BV ratio and 6.0% for the EV/BV ratio. The variation in the log-rank statistic across these parameters and the selected cut-off points is shown in Figure 3.

**Figure 4:**
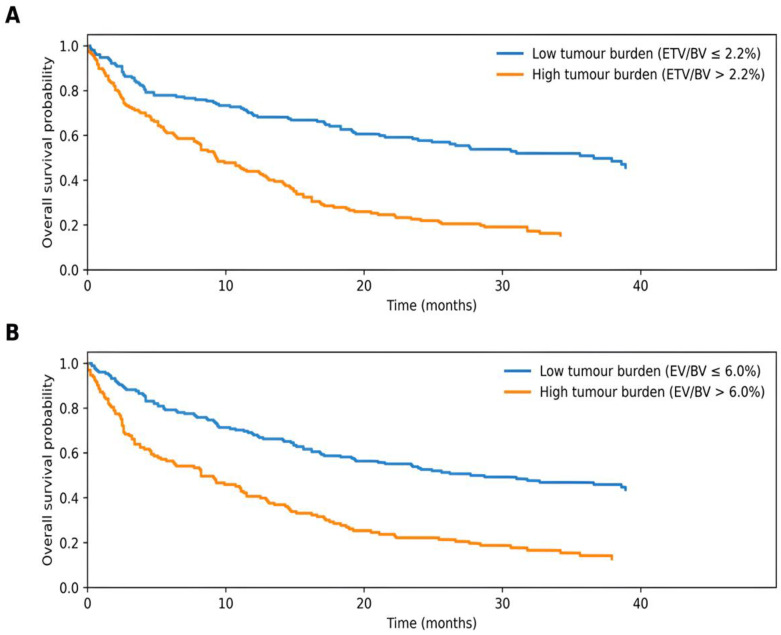
Overall survival curves according to normalized tumor burden. (**A**) Kaplan–Meier survival curves for low and high tumor burden groups defined by the 2.2% cut-off value of the ETV/BV ratio. (**B**) Kaplan–Meier survival curves for low and high tumor burden groups defined by the 6.0% cut-off value of the EV/BV ratio. Overall survival was estimated using the Kaplan–Meier method, and differences between groups were assessed using the log-rank test.

**Figure 5:**
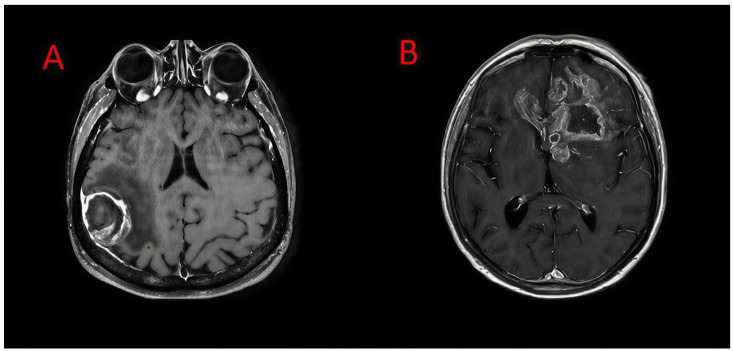
Representative axial contrast-enhanced T1-weighted MRI images demonstrating different levels of tumor burden. (**A**) Lower tumor burden with relatively limited contrast-enhancing lesion. (**B**) Higher tumor burden with more extensive contrast enhancement, associated with deeper structural involvement, contralateral extension, and mass effect.

Using these thresholds, patients were categorized into low and high tumor burden groups. Kaplan–Meier analysis results demonstrated that overall survival was significantly shorter in patients with a higher tumor burden. Based on the ETV/BV ratio, median overall survival was 36.6 months in the low tumor burden group and 9.3 months in the high tumor burden group. Based on the EV/BV ratio, median overall survival was 27.7 months in the low tumor burden group and 8.2 months in the high tumor burden group. Survival curves according to ETV/BV and EV/BV ratios are presented in Figure 4 (log-rank *p* < 0.001). Representative MRI examples of low and high tumor burden are shown in Figure 5.

### 3.4. Univariable Cox Regression Analysis

Univariable Cox regression analysis showed that several variables were associated with overall survival. These included IDH molecular status, age, tumor burden parameters (ETV, ETV/BV, and EV/BV), midline shift, and features reflecting tumor spread. IDH-wildtype status had the strongest association with overall survival (HR 6.863, *p* < 0.001). Tumor burden parameters were also significantly associated with survival, including ETV, ETV/BV (HR 1.987, *p* < 0.001), and EV/BV (HR 1.243, *p* < 0.001). Age, midline shift, deep structure involvement, contralateral extension, multifocality, and ventricular contact were likewise associated with overall survival. Detailed results of the univariable Cox regression analysis are presented in Table 2.

### 3.5. Multivariable Cox Regression Analysis

**Figure 6:**
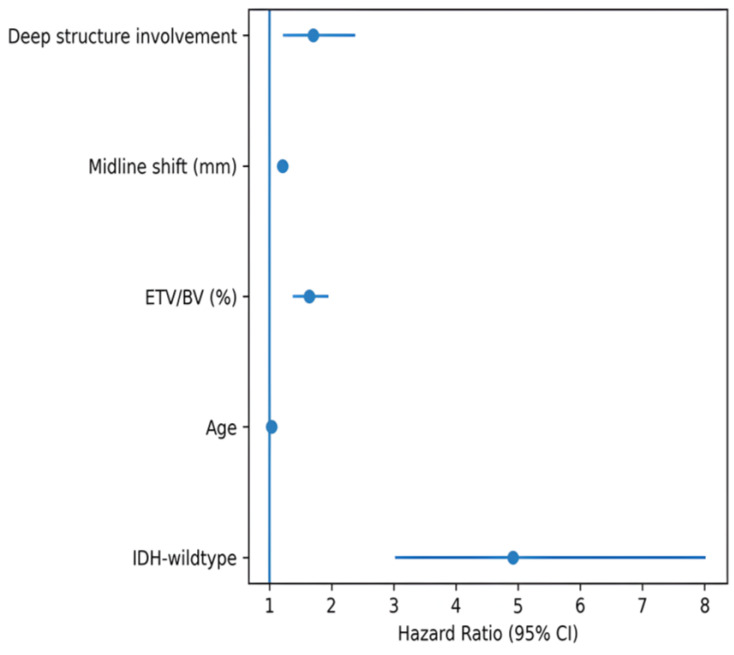
Forest plot showing hazard ratios and 95% confidence intervals of variables independently associated with overall survival in the multivariable Cox regression analysis. The points represent the hazard ratios calculated for each variable, and the horizontal lines represent the 95% confidence intervals. The vertical reference line indicates HR = 1; values to the right of this line indicate increased mortality risk, whereas values to the left indicate decreased mortality risk.

The multivariable Cox regression model included variables identified as significant in the univariable analysis. Consequently, the multivariable analysis results revealed IDH molecular status, age, the ETV/BV ratio, midline shift, and deep structure involvement as independent prognostic indicators of overall survival. IDH-wildtype molecular status was significantly associated with an increased risk of mortality (HR 4.921, 95% CI 3.021–8.017, *p* < 0.001). In addition, higher ETV/BV ratio, age, and midline shift were significantly associated with survival. Deep structure involvement was also identified as an independent prognostic factor. Detailed results of the multivariable Cox regression analysis are presented in Table 3. A forest plot showing the hazard ratios of variables included in the model is presented in Figure 6.

### 3.6. Model Performance

**Figure 7:**
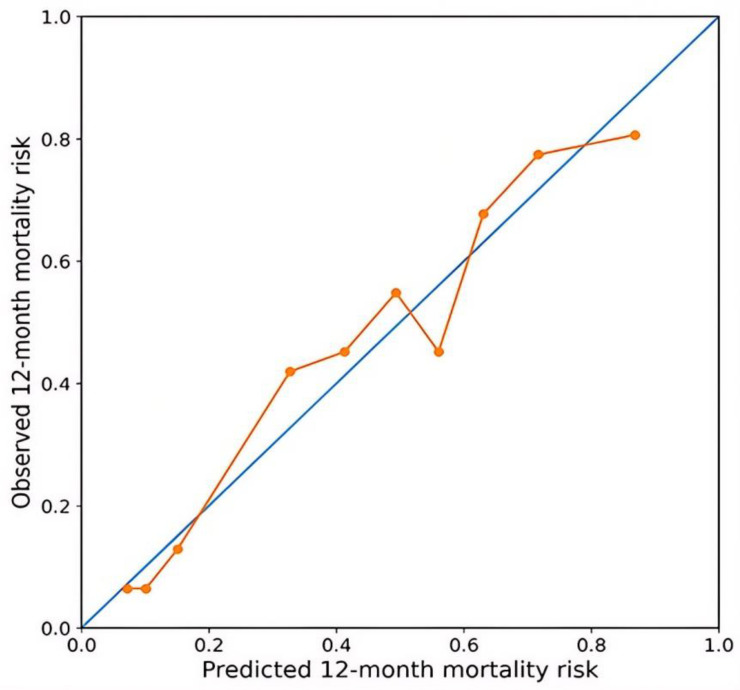
Calibration plot of the multivariable Cox model for 12-month survival. The diagonal line represents ideal agreement between predicted and observed probabilities, and the solid line represents the bootstrap bias-corrected estimates based on 1000 resamples.

Discrimination of the multivariable model was assessed using Harrell’s concordance index (C-index). A clinical model was constructed based on age- and treatment-related variables, and the C-index for this model was 0.69. With the addition of molecular and imaging-based variables, model discrimination improved, and the C-index increased to 0.76. After bootstrap internal validation, the optimism-corrected C-index was calculated as 0.73. The calibration analysis results demonstrated good agreement between predicted and observed survival probabilities at 12 months (Figure 7).

### 3.7. Risk Stratification

**Figure 8:**
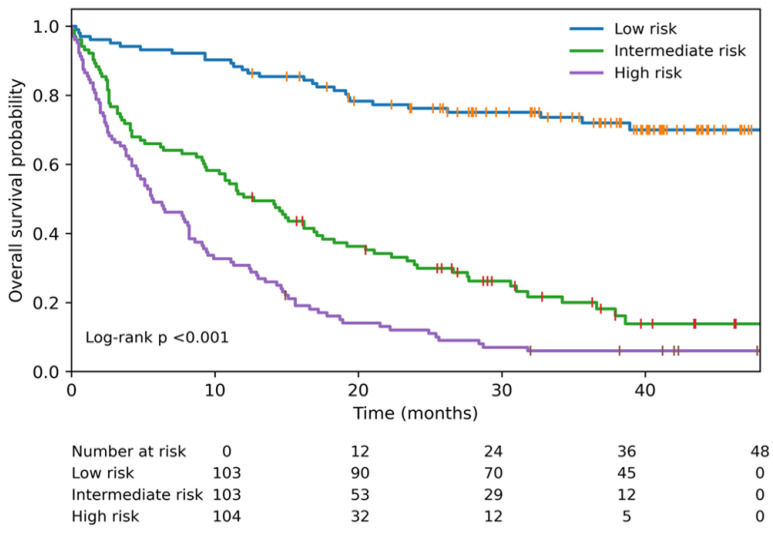
Kaplan–Meier overall survival curves for low-, intermediate-, and high-risk groups defined based on prognostic index values derived from the multivariable Cox regression model. Differences between groups were assessed using the log-rank test.

Using the regression coefficients obtained from the multivariable Cox regression model, an individual prognostic index (risk score) was calculated for each patient. Based on the distribution of prognostic index values, patients were stratified into three groups: low, intermediate, and high risk. Clear differences in overall survival were observed among these risk groups. Median overall survival was not reached in the low-risk group; in comparison, this figure stood at 12.7 months and 5.7 months in the intermediate- and high-risk groups, respectively (log-rank *p* < 0.001). Kaplan–Meier overall survival curves for the risk groups are presented in Figure 8.

### 3.8. Interaction Analysis According to IDH Molecular Status

The potential interaction between radiological variables and IDH status was evaluated. No significant interaction with IDH status was observed for the ETV/BV ratio (*p* = 0.077), midline shift (*p* = 0.612), or deep structure involvement (*p* = 0.910).

## 4. Discussion

In this study, tumor burden was found to be significantly associated with overall survival in grade 4 diffuse astrocytic tumors. In particular, the ETV/BV ratio emerged as an important measure in relation to survival. In the multivariable analysis, the independent factors associated with overall survival were IDH molecular status, age, the ETV/BV ratio, midline shift, and involvement of deep structures, and radiological variables remained significant even when IDH status was included in the model. The association between IDH status and survival observed in our cohort is consistent with its well-established prognostic role in high-grade gliomas. Moreover, incorporating molecular and imaging-based variables improved the prognostic model’s ability to differentiate outcomes, as shown by an increase in the C-index from 0.69 to 0.76. In addition, the model demonstrated acceptable calibration at 12 months, indicating consistency between predicted and observed outcomes.

Tumor burden, a key radiological characteristic, is associated with the prognosis of high-grade gliomas, reflecting the space-occupying effect of the tumor within the brain parenchyma [11,17]. In prior investigations, researchers have predominantly assessed tumor burden through absolute tumor volume, demonstrating a correlation between increased tumor volumes and diminished survival [12,17]. The results of several studies have shown that larger preoperative tumor volume is associated with poorer survival outcomes [11,12]. Nevertheless, given the inter-individual variability in brain volume, an identical tumor volume may reflect disparate levels of tumor burden among patients. Therefore, evaluating tumor volume relative to overall brain volume may provide a more accurate representation of tumor burden [11,14]. In our cohort, absolute tumor volume (ETV) was associated with overall survival; however, this association was not observed after adjustment for additional variables. Accordingly, tumor burden was assessed using the ETV/BV ratio, defined as the proportion of enhancing tumor volume relative to total brain volume. This approach enables a patient-specific evaluation of tumor burden by accounting for interindividual differences in brain volume. Higher ETV/BV ratios were associated with shorter overall survival. Taken together, these results indicate that tumor burden may be more strongly related to prognosis when considered in relation to brain volume rather than as an absolute measure.

Deep structure involvement and midline shift were independently associated with overall survival in our study group, regardless of tumor size. These findings suggest that tumor prognosis may be influenced not only by its size but also by its pattern of spread within the brain. The link between deep structure involvement and reduced survival is in line with previous reports demonstrating shorter survival in glioblastomas with deep supratentorial extension [13,18]. From a surgical perspective, anatomical location and proximity to deep structures may affect the feasibility of resection and, consequently, clinical outcomes, as emphasized in recent reviews [19,20]. Midline shift likewise emerged as an independent prognostic factor. As a marker of mass effect and its influence on intracranial pressure, midline shift may reflect a more advanced local disease burden and a more aggressive tumor profile [15]. In previous studies, researchers likewise reported an association between initial midline shift and shorter survival in patients with glioblastoma, consistent with our findings [15,21]. When considered together, deep structure involvement and midline shift, combined with tumor burden, may represent complementary radiological indicators that more accurately reflect the clinical severity of the disease. No significant interaction was observed between radiological variables and IDH molecular status, indicating that their prognostic effects were similar across IDH subgroups.

The findings of the present study indicate that the integration of imaging-based variables may improve the performance of prognostic models. As models relying solely on clinical variables may have limited prognostic accuracy, the incorporation of radiological parameters reflecting tumor burden, anatomical spread, and mass effect may enable a more comprehensive evaluation of prognosis [8,9,11,16]. Although the role of clinical and molecular variables in glioblastoma prognosis is well established [5], interest in radiological parameters within prognostic models has increased in recent years [8]. Recent studies have shown that volumetric tumor measurements and intracranial tumor spread are associated with survival, indicating a potential role for imaging in prognostic assessment [11,13]. These findings suggest that combining imaging features related to tumor size, anatomical spread, and their effect on surrounding structures may improve the accuracy of prognostic models.

Our findings have clinical implications. Imaging-based parameters provide additional information in the clinical evaluation of patients with glioma. The combined assessment of tumor burden (ETV/BV ratio), deep structure involvement, and midline shift in the preoperative setting supports earlier and more comprehensive prognostic stratification and may assist in surgical planning, defining resection goals, and evaluating adjuvant treatment options. In particular, a higher ETV/BV ratio may reflect a greater tumor burden relative to brain volume and may be taken into consideration during surgical planning and treatment prioritization, while deep structure involvement may influence the feasibility of safe resection, and midline shift may indicate clinically relevant mass effect requiring timely intervention. Such an approach may support clinical decision-making using standard MRI data in the preoperative period.

This study has several strengths that should be acknowledged. The ETV/BV ratio enables tumor burden to be evaluated in a patient-specific manner by accounting for anatomical variability in brain volume. Combining molecular and radiological variables provides a more comprehensive assessment of glioma prognosis. The statistical reliability of the findings is further supported by internal validation using bootstrap resampling. In addition, the relatively large sample size strengthens the analysis. The assessment of discrimination and calibration provides additional evidence for the reliability of the proposed prognostic model. Overall, our results suggest that combining tumor burden with anatomical spread and clinical variables may provide a more informative method to assess prognosis.

Several limitations should also be considered when evaluating the results; due to its retrospective nature and single-center cohort design, selection and information bias may have been introduced, thus limiting the generalizability of the findings. In addition, the prognostic model has yet to be externally validated in an independent cohort. In addition, the use of data-driven cut-off values may introduce optimism bias; however, in the present study, these thresholds were applied exclusively for survival stratification in Kaplan–Meier analyses, whereas primary prognostic modeling was performed using continuous variables within the multivariable Cox regression framework to preserve model robustness and minimize the risk of overfitting. Although volumetric measurements were performed using semi-automated segmentation methods, a degree of observer dependency in image analysis cannot be excluded. Because the imaging data were obtained from routine clinical MRI protocols, differences in acquisition parameters may have affected the measurements. In addition, performance status measures such as the Karnofsky Performance Status were not available for all patients and were, therefore, not included in the analysis. As a result, a residual confounding effect related to performance status cannot be excluded. These factors should be considered when interpreting the results, and it would also be valuable to evaluate the model in independent cohorts. The authors of prospective studies could further clarify how the model performs in daily clinical practice.

## 5. Conclusions

An integrated radiological assessment combining normalized tumor burden, anatomical spread, and mass effect may support prognostic assessment in patients with adult grade 4 diffuse astrocytic tumors, including glioblastoma and IDH-mutant grade 4 astrocytic tumors. In this study, the ETV/BV ratio, midline shift, and deep structure involvement were independently associated with overall survival. The addition of molecular and imaging-based variables was associated with improved model discrimination. Radiological features derived from standard preoperative MRI may help refine preoperative risk stratification. However, this approach requires validation in independent multicenter cohorts before routine use in prognostic modeling.

## Figures and Tables

**Table 1:** Demographic, treatment-related, and radiological characteristics of the study population according to molecular subgroups.

Variable	Overall (n = 310)	IDH-Mutant (n = 96)	IDH-Wildtype (n = 214)	*p*
Age, years (median, IQR)	58 (49–66)	46 (38–55)	62 (54–69)	<0.001
Male sex, n (%)	185 (59.7)	50 (52.1)	135 (63.1)	0.08
Biopsy, n (%)	48 (15.5)	6 (6.2)	42 (19.6)	0.002
Subtotal resection, n (%)	97 (31.3)	28 (29.2)	69 (32.2)	0.61
Gross total resection, n (%)	165 (53.2)	62 (64.6)	103 (48.1)	0.01
Radiotherapy, n (%)	272 (87.7)	90 (93.8)	182 (85.0)	0.03
Temozolomide, n (%)	258 (83.2)	88 (91.7)	170 (79.4)	0.006
Enhancing tumor volume (ETV), cm^3^	31.2 ± 12.3	21.5 ± 9.8	36.9 ± 13.1	<0.001
Edema volume (EV), cm^3^	74.5 ± 28.3	55.2 ± 22.1	86.7 ± 30.5	<0.001
Brain volume (BV), cm^3^	1320 ± 87	1340 ± 82	1310 ± 90	0.09
ETV/BV (%)	2.4 ± 1.0	1.6 ± 0.7	2.9 ± 0.9	<0.001
EV/BV (%)	5.7 ± 2.2	4.1 ± 1.6	6.6 ± 2.2	<0.001
Contralateral extension, n (%)	84 (27.1)	14 (14.6)	70 (32.7)	0.001
Deep structure involvement, n (%)	109 (35.2)	18 (18.8)	91 (42.5)	<0.001
Ventricular contact, n (%)	138 (44.5)	32 (33.3)	106 (49.5)	0.01
Multifocality, n (%)	46 (14.8)	7 (7.3)	39 (18.2)	0.01
Midline shift, mm (median, IQR)	4 (2–7)	3 (1–5)	5 (3–8)	<0.001

Age and midline shift are presented as median (IQR), whereas other continuous variables are presented as mean ± SD. Categorical variables are presented as n (%). *p*-values represent comparisons between IDH-mutant and IDH-wildtype groups. Continuous variables were compared using Student’s *t*-test or the Mann–Whitney U test, as appropriate, and categorical variables using the chi-square or Fisher’s exact test, as appropriate.

**Table 2:** Univariable Cox regression analysis for overall survival.

Variable	HR	95% CI (Lower)	95% CI (Upper)	*p*-Value
ETV (per 1 cm^3^)	1.05	1.04	1.07	<0.001
ETV/BV (%)	1.987	1.725	2.288	<0.001
IDH-wildtype	6.863	4.458	10.564	<0.001
Age	1.050	1.037	1.062	<0.001
Midline shift (mm)	1.310	1.223	1.404	<0.001
EV/BV (%)	1.243	1.173	1.317	<0.001
Deep structure involvement	2.186	1.655	2.889	<0.001
Contralateral extension	1.968	1.477	2.622	<0.001
Multifocality	1.663	1.226	2.255	0.001
Ventricular contact	1.519	1.144	2.017	0.004
Radiotherapy	0.585	0.398	0.858	0.006
Temozolomide	0.661	0.448	0.974	0.036
Surgery: Subtotal resection	1.287	0.976	1.697	0.074
Surgery: Biopsy	1.325	0.899	1.952	0.155

HR: hazard ratio; CI: confidence interval. Univariable analyses were performed using the Cox proportional hazards regression model.

**Table 3:** Multivariable Cox regression analysis for overall survival.

Variable	HR	95% CI (Lower)	95% CI (Upper)	*p*-Value
IDH-wildtype	4.921	3.021	8.017	<0.001
Age	1.032	1.018	1.047	<0.001
ETV/BV (%)	1.641	1.379	1.953	<0.001
Midline shift (mm)	1.214	1.126	1.308	<0.001
Deep structure involvement	1.704	1.219	2.381	0.002

HR: hazard ratio; CI: confidence interval. Multivariable analyses were performed using the Cox proportional hazards regression model.

## Data Availability

The datasets generated and/or analyzed during the current study are available from the corresponding author upon reasonable request. Data cannot be made publicly available due to patient confidentiality and institutional restrictions. Access to anonymized data may be granted to qualified researchers following approval by the Institutional Review Board of Adana City Training and Research Hospital.

## Abbreviations

ETV	Enhancing Tumor Volume
EV	Edema Volume
BV	Brain Volume
IDH	Isocitrate Dehydrogenase
HR	Hazard Ratio
CI	Confidence Interval
ICC	Intraclass Correlation Coefficient
VIF	Variance Inflation Factor
EPV	Events per Variable
SD	Standard Deviation
IQR	Interquartile Range
